# A qualitative transcriptional signature to reclassify histological grade of ER-positive breast cancer patients

**DOI:** 10.1186/s12864-020-6659-0

**Published:** 2020-04-06

**Authors:** Jing Li, Wenbin Jiang, Qirui Liang, Guanghao Liu, Yupeng Dai, Hailong Zheng, Jing Yang, Hao Cai, Guo Zheng

**Affiliations:** 1 Fujian Key Laboratory of Medical Bioinformatics, Department of Bioinformatics, The School of Basic Medical Sciences, Fujian Medical University, Fuzhou, 350122 China; 2 Medical Big Data and Bioinformatics Research Center, First Affiliated Hospital of Gannan Medical University, Ganzhou, Jiangxi China

**Keywords:** Histological grade, ER-positive breast cancer, Gene expression, Survival analysis

## Abstract

**Background:**

Histological grade (HG) is commonly adopted as a prognostic factor for ER-positive breast cancer patients. However, HG evaluation methods, such as the pathological Nottingham grading system, are highly subjective with only 50–85% inter-observer agreements. Specifically, the subjectivity in the pathological assignment of the intermediate grade (HG2) breast cancers, comprising of about half of breast cancer cases, results in uncertain disease outcomes prediction. Here, we developed a qualitative transcriptional signature, based on within-sample relative expression orderings (REOs) of gene pairs, to define HG1 and HG3 and reclassify pathologically-determined HG2 (denoted as pHG2) breast cancer patients.

**Results:**

From the gene pairs with significantly stable REOs in pathologically-determined HG1 (denoted as pHG1) samples and reversely stable REOs in pathologically-determined HG3 (denoted as pHG3) samples, concordantly identified from seven datasets, we extracted a signature which could determine the HG state of samples through evaluating whether the within-sample REOs match with the patterns of the pHG1 REOs or pHG3 REOs. A sample was classified into the HG3 group if at least a half of the REOs of the 10 gene pairs signature within this sample voted for HG3; otherwise, HG1. Using four datasets including samples of early stage (I–II) ER-positive breast cancer patients who accepted surgery only, we validated that this signature was able to reclassify pHG2 patients into HG1 and HG3 groups with significantly different survival time. For the original pHG1 and pHG3 patients, the signature could also more accurately and objectively stratify them into distinct prognostic groups. And the up-regulated and down down-regulated genes in HG1 compared with HG3 involved in cell proliferation and extracellular signal transduction pathways respectively. By comparing with existing signatures, 10-GPS was with prognostic significance and was more aligned with survival of patients especially for pHG2 samples.

**Conclusions:**

The transcriptional qualitative signature can provide an objective assessment of HG states of ER-positive breast cancer patients, especially for reclassifying patients with pHG2, to assist decision making on clinical therapy.

## Background

Breast cancer has the highest incidence and mortality among females [1]. The microscopic morphological assessment of the degree of tumor cell differentiation, represented as tumor histological grades (HGs), has powerful prognostic prediction capability in breast cancer [2–5] and has been incorporated into the eighth edition of American Joint Commission of Cancer staging system [6]. According to the Nottingham grading system, after assessing tubule formation (tubularity), nuclear pleomorphism (nuclearity) and mitotic count, each patient can be assigned to histologic grade 1 (HG1, well-differentiated, slow-growing tumor), histologic grade 2 (HG2, moderately differentiated, slightly faster growing tumor) or histologic grade 3 (HG3, poorly differentiated, highly proliferative tumor) [5]. The higher grade is associated with lower survival rate [3, 4, 7]: the 5-year survival rates of untreated HG1, HG2 and HG3 patients are 95, 75 and 50%, respectively [5, 8, 9]. Considering the excellent prognoses, HG1 patients are amenable for a mild and less harmful anti-cancer therapy. On the contrary, HG3 patients require a more powerful anti-cancer therapy. Genomics analysis indicates that HG1 and HG3 breast carcinomas develop independently along different genetic pathways [10, 11], while HG2 patients (comprising ~ 50% of breast cancer cases) contain a blend of histological features, some of which are common to both HG1 and HG3 tumors, and exhibit a mixed gene expression profiles of HG1 and HG3 [12, 13]. Thus, HG2 breast carcinomas should not be classified as individual HG, but represent clinical and molecular hybrids between HG1 and HG3 diseases [14, 15]. The heterogeneity of the HG2 breast cancers resulted in uncertain disease outcome prediction and there is no standard treatment protocol for clinical decision making [7, 16].

However, the pathological Nottingham grading system, the most employed HG evaluation method, is dependent on adequately prepared hematoxylin-eosin-stained tumor tissue sections to be assessed by an appropriately trained pathologist, which is highly subjective with only 50–85% inter-observer agreements [17–20]. The consensus was even lower for HG2 samples (comprising ~ 50% of breast cancer cases) [15, 21, 22], and it primarily resulted in the unappealing inter-observer agreements among pathologists during evaluating HG. Therefore, many studies have tried to identify transcriptional signatures to reclassify pathologically-determined HG states especially HG2 (pHG2) status of patients in order to improve the therapeutic planning for breast cancer patients [13, 16, 23–25]. However, most of the previously proposed signatures for classifying samples were based on summarized expression measurements of the signature genes, which lack robustness for clinical applications due to widespread batch effects and quality uncertainties of clinical samples [26–29]. The data normalization of samples collected in advance also hinders the feasibility of these signatures in routine clinical practice [16]. In contrast, the qualitative transcriptional signatures based on the within-sample relative expression orderings (REOs) of gene pairs are robust against experimental batch effects [27–29], varied proportions of the tumor epithelial cell in tumor tissues sampled from different tumor locations of the same patient [30], partial RNA degradation during specimen preparation and storage [31] and amplification bias for minimum specimens with about 15–25 cancer cells [32], which are common factors that can lead to failures of quantitative transcriptional signatures in clinical applications. Besides, the qualitative signatures can be applied at the individual level [33]. Based on the within-sample relative expression orderings (REOs) of gene pairs, we have developed prognostic signatures for many cancer types [28, 34–36] and demonstrated their robustness in both inter-laboratories and across-platforms tests [29, 37].

Approximately 70% breast cancer patient express estrogen receptor (ER) according to American Cancer Society [1], adjuvant endocrine therapy is the routine regimen, and only for ER-positive patients with high HG, combined chemotherapy is suggested. It has been reported that there is a significant transcriptional difference between ER-positive and ER-negative cohorts [38]. ER-positive status is associated with a heterogeneous mixture of histologic grades, whereas ER-negative status is generally associated with HG3 [39].

In this study, we aimed to develop a qualitative transcriptional signature to identify HG states objectively in ER-positive breast cancers. Using gene expression profiles of 932 ER-positive early stage breast cancer patients, we developed a qualitative signature to allocate each patient into the pathologically-determined HG1 (denoted as pHG1) or HG3 (denoted as pHG3) group. Using four independent validation datasets including a total 524 samples of ER-positive breast cancer patients who accepted surgery only, the signature could find out a certain percentage of pHG1 patients as HG3 patients with worse prognoses and some pHG3 patients identified as HG1 patients with better prognoses. Especially, we adopted an objective approach to validate the signature through evaluating whether the pHG2 patients reclassified as HG1 had better prognoses than the pHG2 patients reclassified as HG3.

## Results

### Development of the REO-based grade signature

**Fig. 1:**
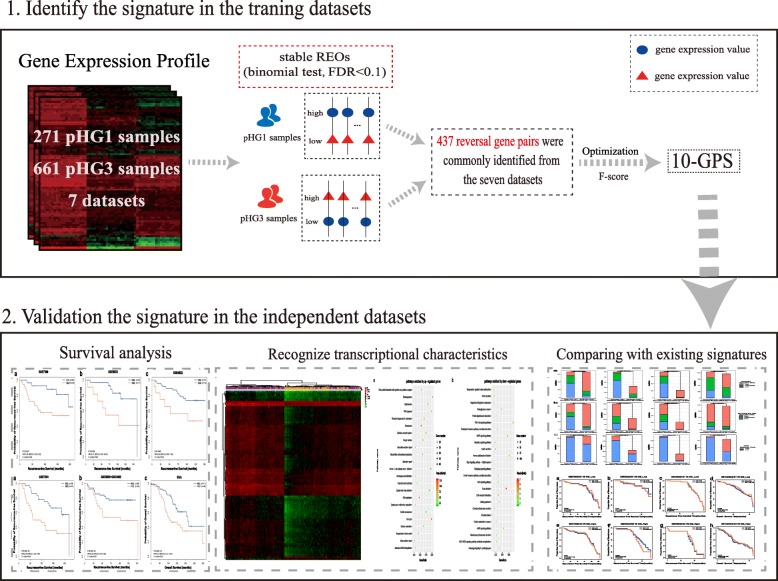
Workflow for identification and evaluation of the qualitative signature of reclassifying histological grade status. The workflow has 2 major analysis steps: (1), identification the qualitative signature in surgery only samples and (2), evaluation of the qualitative signature in surgery only samples and TCGA dataset

The flowchart of this study was described in Fig. 1. For each of the seven training datasets (Table 1), with FDR < 0.1, we firstly identified gene pairs with significantly stable REOs in the pHG1 and pHG3 groups, respectively, and then identified gene pairs with reversal REOs between the two groups. Then, 437 gene pairs were commonly identified from the seven datasets and they consistently showed the same reversal REO patterns between the pHG1 and pHG3 groups in the seven datasets. Next, we performed a forward-stepwise selection procedure to search a set of gene pairs that achieved the highest F-score according to the classification rule as follows: a sample was classified into the HG3 group if at least a half of the REOs of the set of gene pairs within the sample voted for HG3; otherwise, into the HG1 group. Finally, we obtained 10 gene pairs, denoted as 10-GPS (Table 2), to distinguish different histological grades with the highest F-score (0.8884). In the training data, the apparent specificity for HG1 samples was 90.77% and the apparent sensitivity for HG3 samples was 86.99%. The performance of the transcriptional grade signature in each training dataset can be found in Additional file 1: Table S1. Notably, the apparently imperfect performance should be reasonable because HG evaluation based on the pathological Nottingham grading system is highly subjective with only 50–85% inter-observer agreements [17–20]. We speculated that the 10-GPS could provide a more objective and clinically relevant measure of tumor grade with prognostic significance.

**Table 1:** Description of the datasets used in this study

Datasets	Platform	HG1	HG2	HG3	#Genes
Training datasets					
GSE19615	Affymetrix array	23	–	25	20,486
GSE21653	Affymetrix array	37	–	47	20,486
GSE1456	Affymetrix array	26	–	40	12,432
GSE3494	Affymetrix array	62	–	33	12,432
EGAD00010000210^a^	Illumina beadchip	30	–	221	25,186
EGAD00010000211^a^	Illumina beadchip	35	–	125	25,186
TCGA	Illumina HiSeq 2000	58	–	170	20,720
Validation datasets					
GSE7390	Affymetrix Array	29	68	35	12,432
GSE6532	Affymetrix Array	29	31	12	12,432
GSE4922	Affymetrix Array	48	60	11	12,432
EGA^b^	Illumina beadchip	35	120	46	25,186

Note: ^a^ denoted dataset provided by the Molecular Taxonomy of Breast Cancer International Consortium (METABRIC) [40]. EGA^b^ denoted dataset integrated from EGAD00010000210 and EGAD00010000211

**Table 2:** The REO-based transcriptomic grade signature

Gene A		Gene B	
Gene ID	Gene symbol	Gene ID	Gene symbol
80,127	BBOF1	9319	TRIP13
22,885	ABLIM3	24,137	KIF4A
1848	DUSP6	11,065	UBE2C
9486	CHST10	9319	TRIP13
11,122	PTPRT	9833	MELK
6271	S100A1	9319	TRIP13
23,403	FBXO46	8140	SLC7A5
23,303	KIF13B	27,346	TMEM97
1101	CHAD	11,004	KIF2C
51,310	SLC22A17	9212	AURKB

Note: Gene A has a higher expression level than Gene B in HG1 groups

**Fig. 2:**
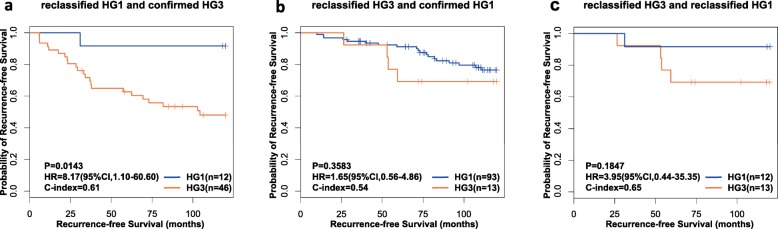
Kaplan–Meier estimates of relapse-free survival in dataset merged from GSE7390, GSE6532 and GSE4922. **a** Relapse-free survival curves for reclassified HG3 and confirmed HG1 breast cancer patients. **b** Relapse-free survival curves for reclassified HG1 and confirmed HG3 breast cancer patients. **c** Relapse-free survival curves for reclassified HG3 and reclassified HG1 breast cancer patients

We validated the above speculation based on the knowledge that HG3 patients were with lower survival rate than HG1 patients [3, 7]. Here, we collected another four independent datasets (Table 1) including samples with RFS or OS data of early stage ER-positive breast cancer patients who accepted surgery only. When the 10-GPS was applied to these datasets, the averaged apparent sensitivity for all HG3 samples was 83.1% and the average apparent specificity for all HG1 samples was 78.4%. In a merged dataset from the three validation datasets with the RFS information, the 12 pHG3 patients reclassified as HG1 by the signature had significantly higher 10-years RFS rate than that of the 46 HG3 patients confirmed by the signature (*p* = 0.0143; HR = 8.17, 95% CI: 1.10–60.60; C-index = 0.61, Fig. 2a). And, we also compared 10-years RFS rates between the 13 pHG1 patients reclassified as HG3 by the 10-GPS and the 93 HG1 patients confirmed by the 10-GPS. Despite no statistical difference was, there was trend to be different between the two groups (*p* = 0.3583; HR = 1.65, 95% CI: 0.56–4.86; C-index = 0.54, Fig. 2b). There was also trend of difference between the RFS rate of 12 pHG3 patients reclassified as HG1 by the signature and that of 13 pHG1 patients reclassified as HG3 by the 10-GPS (*p* = 0.1847; HR = 3.95, 95% CI: 0.44–35.35; C-index = 0.65, Fig. 2c).

**Fig. 3:**
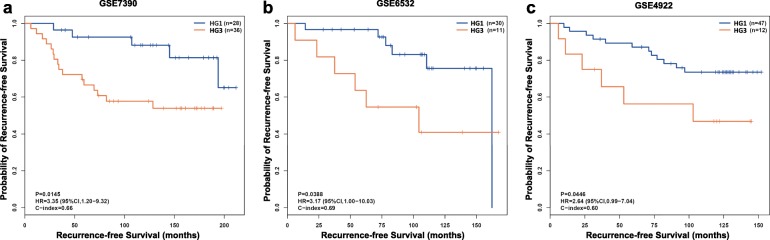
Kaplan–Meier estimates of relapse-free survival. **a** Relapse-free survival curves for HG1 and HG3 breast cancer patients in dataset GSE7390. **b** Relapse-free survival curves for HG1 and HG3 breast cancer patients in dataset GSE6532. **c** Relapse-free survival curves for HG1 and HG3 breast cancer patients in dataset GSE4922

In each of the three validation datasets with the RFS information, we also compared the survival between the pHG1 and pHG3 patients diagnosed by the pathological Nottingham grading system and the survival between the HG1 and HG3 patients reclassified by the 10-GPS from the pHG1 and pHG3 patients. Significant difference of RFS between the pHG1 and pHG3 patients was observed only in GSE6532 and GSE4922 dataset (Additional file 2: Fig. S1). However, the HG1 patients showed significantly better RFS than those of HG3 patients in all the three datasets (Fig. 3). No significant difference of prognosis was observed between pHG1 and HG1 cohorts for each of the four validation datasets. Similar for pHG3 and HG3 cohorts for each of the four datasets (Additional file 3 Fig. S2). The majority histological grade labels of pHG1 and pHG3 sample were correct, which resulting no significant difference of survival mentioned above. These results demonstrated that the 10-GPS can more accurately and objectively stratify samples into distinct prognostic groups.

### Application of the signature to reclassification of HG2 samples

Then, we used the 10-GPS to reclassify the pHG2 samples of the above four validation datasets with RFS or OS information (Table 1) into the HG1 and HG3 groups, respectively, and evaluated their prognostic differences.

**Fig. 4:**
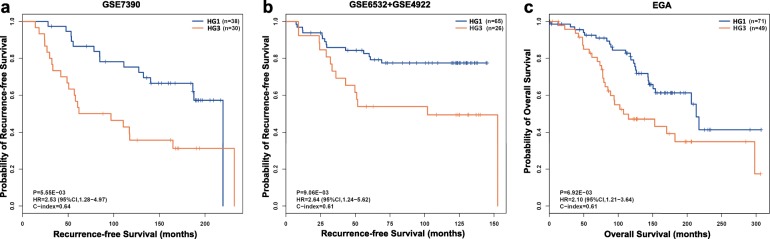
Kaplan–Meier estimates of survival. **a** Relapse-free survival curves for HG1 and HG3 patients reclassified from pHG2 breast cancer patients in dataset GSE7390. **b** Relapse-free survival curves for HG1 and HG3 patients reclassified from pHG2 breast cancer patients in dataset merged from GSE6532 and GSE4922. **c** Overall survival curves for HG1 and HG3 patients reclassified from pHG2 breast cancer patients in dataset EGA

Firstly, for the 68 pHG2 samples of the GSE7390 dataset, the 10-GPS signature allocated 38 and 30 patients into the HG1 and HG3 groups, respectively. And, the former ones had significantly higher RFS rate than the latter ones (*p* = 5.55E-03; HR = 2.53, 95% CI: 1.28–4.97; C-index = 0.64; Fig. 4a). Then, in the 91 pHG2 patients combined from the datasets of GSE6532 and GSE4922 with small sample sizes, the RFS rate of the 65 patients stratified into the HG1 group was significantly higher than that of the 26 patients stratified into the HG3 group (*p* = 9.06E-03; HR = 2.64, 95% CI: 1.24–5.62; C-index = 0.61; Fig. 4b). In the EGA dataset, the 71 HG1 patients classified by the 10-GPS also displayed significant higher OS rate than that of the 49 HG3 patients classified by the 10-GPS (*p* = 6.92E-03; HR = 2.10, 95% CI: 1.21–3.64; C-index = 0.61; Fig. 4c).

### Transcriptional characteristics of the low-HG and high-HG samples recognized by the 10-GPS

**Fig. 5:**
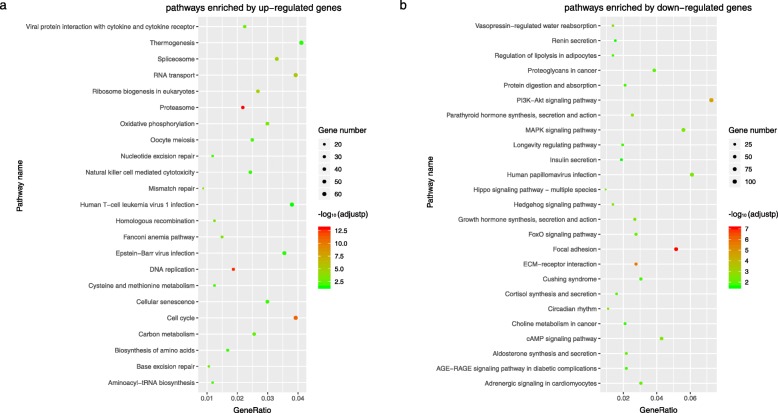
Functional pathways enriched with differential expressed genes between HG1 and HG3 groups. **a** Pathways enriched by up-regulated genes. **b** Pathways enriched by down-regulated genes

**Fig. 6:**
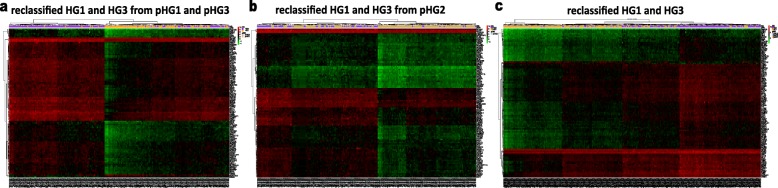
Heatmap of differential expressed genes identified from HG1 and HG3 samples reclassified from (**a**) pHG1 and pHG3 samples. **b** pHG2 samples (**c**) all pathological grade samples in TCGA along with hierarchical clustering. The bottom list indicated the sample names and the right list indicated the differential expressed genes. The three color-coded bars right the heatmap indicated expression value normalized by log2 (left, red indicated up-regulated and green indicated down-regulated), pathological histological grade (upper right) and reclassified histological grade (lower right)

In the TCGA-BRCA dataset, we used the limma algorithm and found 6194 differentially expressed genes (DEGs) between the 58 pHG1 and 170 pHG3 samples diagnosed by the pathological Nottingham grading system (FDR < 0.05, Additional file 4: Table S2). Applying the 10-GPS to these samples, 94 samples were allocated into the HG1 group and the other 134 samples were allocated into the HG3 group. We identified 8087 DEGs between the two reclassified groups with the same FDR control (Additional file 4: Table S3). And of these genes, up-regulated genes were significantly associated with proliferation and down-regulated were significantly associated with extracellular signal transduction (Fig. 5). When comparing the two DEG lists, we found that 5472 (88.34%) of the 6194 DEGs between the original HG1-HG3 groups were also included in the DEGs identified after sample reclassification and the dysregulation directions of the overlapped genes reached up to 100% (binomial test, *p* < 1.10E–16). We also identified 3126 DEGs between 145 HG1 (denoted as LHG2) samples and 71 HG3 (denoted as HHG2) samples recognized from the pHG2 samples with the aid of 10-GPS (Additional file 4: Table S4). About 31.14% of the 2519 DEGs were also included in the 8087 DEGs. The concordance score of the 1164 overlapped DEGs was 99.92%, which was unlikely to happen by chance (binomial test, p < 1.10E–16). Moreover, after reclassifying HG status of samples for TCGA dataset, we identified differential expressed genes identified from pHG1 and pHG3 samples, from HG1 and HG3 samples reclassified from pHG2 samples, and HG1 and HG3 samples reclassified from overall samples respectively (Fig. 6). In all of the three two-way clustering heatmaps in Fig. 6, each of the two reclassified histological grade sample subclasses (two child nodes under the root node in sample clustering tree) contained both HG1 and HG3 samples although HG1 (or HG3) samples were in the majority. This may be caused by the difference between quantitative and qualitative expression relationship essentially. After identified enriched pathway lists by differential expressed genes for all the four datasets, the most common pathways were associated with proliferation and extracellular signal transduction (Additional file 5: Fig. S3) The clearer transcriptional differences between the two reclassified groups indicated that the 10-GPS could more accurately and objectively stratify samples into distinct histological grade groups.

### Comparison of 10-GPS prognostic significance with GGI, Oncotype DX and PAM50

**Fig. 7:**
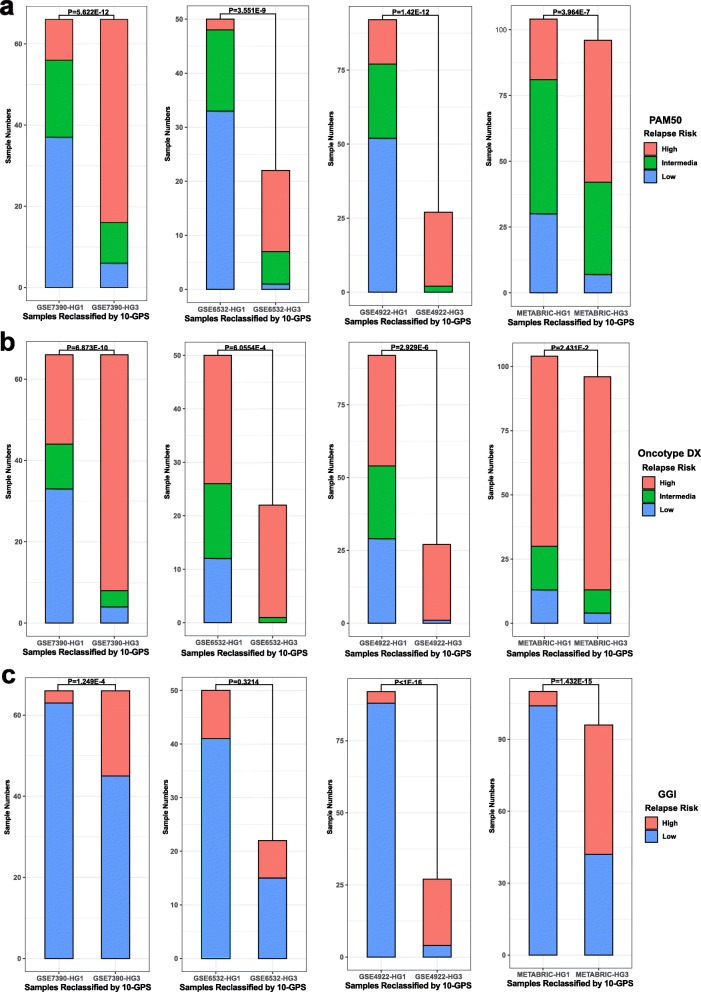
Composition of samples for HG1 and HG3 groups in four validation datasets by comparing with relapse risk identified by (**a**) PAM50. **b** Oncotype DX and (**c**) GGI

**Fig. 8:**
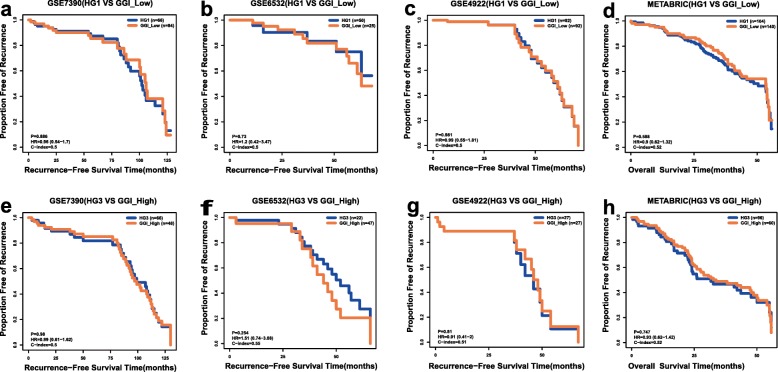
Kaplan–Meier estimates of survival (**a**-**c**) Relapse-free survival curves for low-risk group identified by GGI and HG1 group identified by 10-GPS in GSE7390, GSE6532, GSE7390 (**d**) Overall survival curves for low-risk group identified by GGI and HG1 group identified by 10-GPS in METABRIC (**e**-**g**) Relapse-free survival curves for high-risk group identified by GGI and HG3 group identified by 10-GPS in GSE7390, GSE6532, GSE7390 (**h**) Overall survival curves for high-risk group identified by GGI and HG3 group identified by 10-GPS in METABRIC\

For each of the four validation datasets, we obtained relapse risk by using existing signatures such as Gene expression Grade Index (GGI) [41], PAM50 [42] and Oncotype DX [43]. Chi-square test were conducted, revealing that there were significant differences in relapse risks generated by almost all the three existing signatures between HG1 and HG3 cohorts (Fig. 7, Additional file 1: Table S5). This indicating that histological grade status reclassified by 10-GPS were with prognostic significance. Meanwhile, no prognostic differences between low-risk group identified by GGI and HG1 group identified by 10-GPS were observed (Fig. 8a-d). It was similar for high-risk group identified by GGI and HG3 group identified by 10-GPS were observed (Fig. 8e-h). Significant prognostic difference between HG1 samples who were identified as low-risk by GGI and HG1 samples who were identified as high-risk by GGI was observed only in GSE4922, which might result from unbalanced samples (Additional file 6 Fig. S4 a-d). No significant prognostic difference between HG3 samples who were identified as low-risk by GGI and HG3 samples who were identified as high-risk by GGI was observed (Additional file 6 Fig. S4 e-h). It showed that the prognostic significance of 10-GPS was more aligned with the survival of patients. Moreover, HG1 samples who were identified as high-risk and HG3 samples who were identified as low-risk were mainly from pHG2 cohort. The consistency between the result and prior knowledge that pHG2 are with low inter-observer agreements was reasonable. It implied that 10-GPS was feasible for reclassifying histological grade status, especially for pHG2 samples. All these comparisons indicated that 10-GPS could effectively reclassify into distinct histological grade groups with significantly prognostic difference.

## Discussion

In this study, we developed a histological grade signature consisting of 10 gene pairs (10-GPS) to reclassify the ER-positive breast cancer patients to distinct prognostic groups. This transcriptional qualitative signature, which is based on REOs in an individual sample, was highly robust against experimental batch effects, varied proportions of the tumor epithelial cell in tumor tissues [30], RNA degradation [31], and amplification bias for minimum specimens [32]. All of these merits make it possible to apply the 10-GPS into clinical practices. The 10-GPS could not only objectively and accurately allocate HG1 and HG3 patients but also reclassify HG2 patients into two groups with significantly different survival rates. For clinical application, the patients allocated into the HG3 group should receive adjuvant chemotherapy followed by endocrine therapy; and the patients allocated into the HG1 group were recommended the endocrine therapy only.

Fortunately, based on the working assumption that the majority labels of the pHG1 and pHG3 samples were right, thus we employed a supervised learning method to develop the signature. Imperfect F-score of 0.8884 just suggested that the 10-GPS did not over-fit the training dataset. There’s no surprise that the apparent sensitivity for all HG3 samples was 83.10% and the apparent specificity for all HG1 samples was 78.40% in the validation datasets. In this study, we adopted a more objective approach to validate the signature through evaluating whether the reclassified HG1 patients could have better prognosis than that of HG3 patients. In four independent validation datasets, the reclassified HG1 and HG3 groups recognized by the 10-GPS from the original HG2 patients or from the original HG1 and HG3 patients had significantly different survival.

We expected that the 10-GPS can replace or serve as auxiliary reference of the pathological Nottingham grading system to stratify ER-positive patients into two distinct groups in clinical practices. When applying the 10-GPS to all 132 samples of the GSE7390 dataset, the grade signature classified 66 patients into the HG1 group and 66 patients into the HG3 group. The RFS rate of the former group was significantly higher than that of the latter group (*p* = 7.74E-04; HR = 2.49, 95% CI: 1.44–4.33; C-index = 0.64; Additional file 7: Fig. S5 a). Significantly different survival time between HG1 and HG3 groups reclassified by the 10-GPS were also observed in another three independent validation datasets (Additional file 7: Fig. S5 2b-d). By comparing with existing signatures, 10-GPS was with prognostic significance and was more aligned with survival of patients especially for pHG2 samples.

Some signatures had been developed to re-classify the HG status of BC samples, for example, during the development of GGI, samples with ≥100 ng and a RIN ≥ 7 were considered as qualified, and the quantitative threshold has been adopted during the re-classification process which might be affected by constituent ratios of samples [41], which results lacking of reproducibility for datasets generated by different labs or platforms and limitation of individual application. Meanwhile, the qualitative signature 10-GPS is with wider application to trace samples [32], samples with lower RIN [31] and samples with low tumor-purity [30].

A limitation of this study is that we were unable to directly evaluate the signature in RNA-sequencing, such as those archived in the TCGA database, where no patients accepted surgery only and sizes of samples who accepted same therapy regimen were too small to perform the validation. Here, we only indirectly validate the 10-GPS in RNA-seq data of the TCGA-BRCA dataset through analysis of DEGs between the HG1 and HG3 groups. Another limitation of our study is no evidence could prove that application of the signature to patients who have undergo chemotherapy or radiation therapy is feasible (Additional file 8 Fig. S6). In the future, we will evaluate the performance of the signature developed in this study for expression data produced by RNA-sequencing or PCR platforms and evaluate applying the signature to patients who have undergo chemotherapy or radiation therapy.

## Conclusions

Pathological histological grade evaluation methods are with high subjectivity, especially for the evaluation of HG2 breast cancer specimens. The transcriptional qualitative signature is objective for the evaluation and is robust for application of microscale samples, samples with lower RIN and samples with low tumor-purity, and can assist making on clinical therapy especially for patients with pHG2.

## Methods

### Data collection and pre-processing

We collected gene expression profiles of 932 ER-positive breast cancer samples with pHG1 or pHG3 diagnosed by the pathological Nottingham grading system. To evaluate whether the reclassified two groups have significantly different survival time, we also collected independent expression data of 524 early stage (I–II) ER-positive breast cancer patients who accepted surgery only. All the breast cancer datasets used in this study were summarized in Table 1. The overall pathological histologic grades of TCGA samples were obtained from the study of Zheng Ping et al. [44].

For Affymetrix array data, raw intensity files (.cel), downloaded from the Gene Expression Omnibus database (GEO, https://www.ncbi.nlm.nih.gov/geo), were processed with the Robust Multichip Average algorithm (RMA) algorithm for background adjustment without quantile normalization [45]. For Illumina beadchip data, the normalized expression data under accession number EGAD00010000210 and EGAD00010000211 [40] were downloaded from the European Genome-Phenome Archive (http://www.ebi.ac.uk/ega/). When processing the data of the two platforms, each probe set ID was mapped to Gene ID according the corresponding annotation files, and then probe sets that mapped to multiple Gene IDs or did not map to any Gene ID were removed. The expression measurements of all probe sets corresponding to the same Gene ID were averaged to obtain a single measurement (on the log2 scale). For RNA-Seq data downloaded from The Cancer Genome Atlas database (TCGA, https://www.cancer.gov/tcga), the level 3 Fragments per Kilobase of transcript per Million mapped reads (FPKM) [46] values were downloaded from The Cancer Genome Atlas (TCGA) database. After removing genes with a count of 0 in more than 75% of samples, other zero values were filled with the smallest count in this expression data. The Ensembl gene IDs corresponding to the unique Entrez gene IDs were used. From the seven training datasets, we extracted expression profiles of 11,587 genes commonly measured by the three platforms (Affymetrix array, Illumina beadchip and Illumina HiSeq 2000) for subsequent analysis.

### Development of the transcriptional signature for histological grade

Firstly, we identified the significantly stable REOs in pHG1 groups of each training dataset. For a given gene pair (*G*_*i*_, *G*_*j*_), if the REO pattern (*G*_*i*_ > *G*_*j*_ or *G*_*i*_ < *G*_*j*_) was kept in more samples than expected by random chance, we defined the REO pattern of this gene pair, *G*_*i*_ > *G*_*j*_ in pHG1 group (or equally *G*_*i*_ < *G*_*j*_ in pHG3 group) as a stable REO characterizing pHG1 samples. The significance of the REO pattern is determined by a binomial test [47] as follows,

1 $$ P=1-\sum \limits_{i=0}^{s-1}\left(\begin{array}{c}n\\ {}i\end{array}\right){\left({p}_0\right)}^i{\left(1-{p}_0\right)}^{n-1} $$

where *s* is the number of samples in which gene *i* has a higher (or lower) expression level than gene *j* in a total of *n* samples, *p*_0_ is the probability of observing a certain REO pattern (*G*_*i*_ > *G*_*j*_ or *G*_*i*_ < *G*_*j*_) in a sample by chance (*p*_0_ = 0.5). The Benjamini-Hochberg multiple testing correction was used to estimate the false discovery rate (FDR) [48]. Then, we identified the gene pairs with stable REOs in pHG3 group but reversal REO patterns between the pHG1 and pHG3 groups in each training dataset.

After selecting gene pairs with concordant reversal REOs among the seven training datasets, a forward-stepwise selection algorithm was performed to search for optimal subset of these gene pairs that resulted in the highest F-score. The F-score, harmonic mean of sensitivity and specificity, was calculated as follows,

2 $$ \mathrm{F}-\mathrm{score}=\frac{2\times \mathrm{sensitivity}\times \mathrm{specificity}}{\mathrm{sensitivity}+\mathrm{specificity}} $$

where sensitivity was defined as the proportion of correctly identified HG3 samples among all pHG3 samples, and specificity was defined as the proportion of correctly identified HG1 samples among all pHG1 samples.

### Survival analysis

Recurrence-free survival (RFS) and overall survival (OS) served as the prognosis endpoint. Kaplan-Meier survival plots and log-rank tests [49] were used to evaluate the differences in RFS and OS of distinct groups. The Cox proportional-hazards model was also performed to calculate the hazard ratios (HRs) and their 95% confidence intervals (CIs) [50]. To evaluate the predictive performance of a signature we also adopted the concordance index (C-index), which is a measure of overall concordance between predicted risk scores and observed survival [51, 52].

### Differential expression and functional enrichment analysis

After using limma package in R, the expression values of all tumor samples of TCGA-BRCA dataset were log-transformed by voom and the batch effects such as plate was corrected by removeBatchEffect. Then differential expressed genes were identified. The Fisher [53] was used to determine the significance of biological pathways enriched with a set of interested genes by hypergeometric distribution test.

## Supplementary information

**Additional file 1: Table S1.** The performance of the transcriptional grade signature in each training dataset, shown with apparent specificity, sensitivity and F-score. Table S5. Comparison of prognostic risks between 10-GPS and PAM50 (or Oncotype DX, GGI).

**Additional file 2: Fig. S1.** Kaplan–Meier estimates of survival. (a-c) Relapse-free survival curves for pHG1 and pHG3 patients in dataset GSE7390, GSE6532 and GSE4922. (d) Overall survival curves for pHG1 and pHG3 patients reclassified from all breast cancer patients in dataset EGA.

**Additional file 3: Fig. S2.** Kaplan–Meier estimates of survival. (a-c) Relapse-free survival curves for pHG1 and HG1 patients reclassified from all breast cancer patients in dataset GSE7390, GSE6532 and GSE4922 (d) Overall survival curves for pHG1 and HG1 patients reclassified from all breast cancer patients in dataset EGA. (e-g) Relapse-free survival curves for pHG3 and HG3 patients reclassified from all breast cancer patients in dataset GSE7390, GSE6532 and GSE4922 (h) Overall survival curves for pHG3 and HG3 patients reclassified from all breast cancer patients in dataset EGA.

**Additional file 4: Table S2–4** Differential expressed gene identified in TCGA dataset. Table S2. Differential expressed gene identified in pHG1 and pHG3 samples. Table S3. Differential expressed gene identified in HG1 and HG3 samples reclassified from pHG1 and pHG3 samples. Table S4. Differential expressed gene identified in HG1 and HG3 samples reclassified from all samples.

**Additional file 5: Fig. S3.** Reproducibility of pathways enriched by differential expressed genes identified in TCGA dataset. The number of times each pathway enriched in the four validation datasets was ranged from 0 to 4. The more positive the number, the deeper the red color of horizontal bar.

**Additional file 6: Fig. S4.** Kaplan–Meier estimates of survival (a-c) Relapse-free survival curves for low-risk and high -risk group identified by GGI in HG1 group identified by 10-GPS in GSE7390, GSE6532, GSE4922 (d) Overall survival curves for low-risk and high -risk group identified by GGI in HG1 group identified by 10-GPS in METABRIC (e-g) Relapse-free survival curves for low-risk and high -risk group identified by GGI in HG3 group identified by 10-GPS in GSE7390, GSE6532, GSE4922 (h) Overall survival curves for low-risk and high -risk group identified by GGI in HG3 group identified by 10-GPS in METABRIC.

**Additional file 7: Fig. S5.** Kaplan–Meier estimates of survival. (a) Relapse-free survival curves for HG1 and HG3 patients reclassified from all breast cancer patients in dataset GSE7390. (b) Relapse-free survival curves for HG1 and HG3 patients reclassified from all breast cancer patients in dataset GSE6532. (c) Relapse-free survival curves for HG1 and HG3 patients reclassified from all breast cancer patients in dataset GSE4922. (d) Overall survival curves for HG1 and HG3 patients reclassified from all breast cancer patients in dataset EGA.

**Additional file 8: Fig. S6.** Kaplan–Meier estimates of survival. Relapse-free survival curves for HG1 and HG3 patients reclassified from all breast cancer patients in dataset GSE16391.

## Data Availability

Previous data analyzed in this study should be requested from the authors of the original publications. Please see methods cohort description (Table 1), for references to these publications. The Affymetrix array data (GSE19615, GSE21653, GSE1456, GSE3494, GSE7390, GSE6532 and GSE4922) that support the findings of this study were downloaded from Gene Expression Omnibus database (GEO, https://www.ncbi.nlm.nih.gov/geo). The Illumina beadchip datasets (EGAD00010000210 and EGAD00010000211) that support the findings of this study were downloaded from the European Genome-Phenome Archive (http://www.ebi.ac.uk/ega/) after permitted by the Molecular Taxonomy of Breast Cancer International Consortium (METABRIC) for non-commercial use. The RNA-Seq data (TCGA) that support the findings of this study were downloaded from The Cancer Genome Atlas database (TCGA, https://www.cancer.gov/tcga).

## Abbreviations

HG: histological grade
pHG: pathologically-determined histological grade
C-index: concordance index
CIs: confidence intervals
HRs: hazard ratios
FDR: false discovery rate
REOs: relative expression orderings
RMA: Robust Multi-Array Average
