# Supplementary material for: A qualitative transcriptional signature to reclassify histological grade of ER-positive breast cancer patients
Source: BMC Genomics. 2020 Apr 6;21:283. doi: 10.1186/s12864-020-6659-0 (PMC7132979; doi:10.1186/s12864-020-6659-0)
Supplement: Supplementary file 1 — Additional file 1: Table S1. The performance of the transcriptional grade signature in each training dataset, shown with apparent specificity, sensitivity and F-score. Table S5. Comparison of prognostic risks between 10-GPS and PAM50 (or Oncotype DX, GGI). [file 12864_2020_6659_MOESM1_ESM.docx]

Table S1. The apparent performance of 10-GPS in each of the train datasets

| **Datasets** | **Specificity** | **Sensitivity** | **F-score** |
| --- | --- | --- | --- |
| GSE19615 | 82.61% | 80.00% | 81.28% |
| GSE21653 | 86.49% | 91.49% | 88.92% |
| GSE1456 | 96.15% | 87.50% | 91.62% |
| GSE3494 | 91.94% | 87.88% | 89.86% |
| EGA_210 | 96.67% | 82.35% | 88.94% |
| EGA_211 | 97.14% | 89.60% | 93.22% |
| TCGA_BRCA | 86.21% | 90.59% | 88.34% |

Table S5. Comparison of prognostic risks between 10-GPS and PAM50 (or Oncotype DX, GGI)

|  | | **10-GPS** | | | | | | | |
| --- | --- | --- | --- | --- | --- | --- | --- | --- | --- |
|  |  | **GSE7390** | | **GSE6532** | | **GSE4922** | | **METABRIC** | |
|  |  | HG1 | HG3 | HG1 | HG3 | HG1 | HG3 | HG1 | HG3 |
| **PAM50** | High | 10 | 50 | 2 | 15 | 15 | 25 | 23 | 54 |
|  | Intermedia | 19 | 10 | 15 | 6 | 25 | 2 | 51 | 35 |
|  | Low | 37 | 6 | 33 | 1 | 52 | 0 | 30 | 7 |
| ***P*** | | 5.622E-12 | | 3.551E-9 | | 1.142E-12 | | 3.964E-7 | |
| **Oncotype DX** | High | 22 | 58 | 24 | 21 | 38 | 26 | 74 | 83 |
|  | Intermedia | 11 | 4 | 14 | 1 | 25 | 0 | 17 | 9 |
|  | Low | 33 | 4 | 12 | 0 | 29 | 1 | 13 | 4 |
| ***P*** | | 6.873E-10 | | 6.055E-4 | | 2.929E-6 | | 2.431E-2 | |
| **GGI** | High | 3 | 21 | 9 | 7 | 4 | 23 | 6 | 54 |
|  | Low | 63 | 45 | 41 | 15 | 88 | 4 | 104 | 42 |
| ***P*** | | 1.249E-4 | | 0.3214 | | <1E-16 | | 4.132E-15 | |
