# Supplementary figures and images for: A qualitative transcriptional signature to reclassify histological grade of ER-positive breast cancer patients
Source: BMC Genomics. 2020 Apr 6;21:283. doi: 10.1186/s12864-020-6659-0 (PMC7132979; doi:10.1186/s12864-020-6659-0)

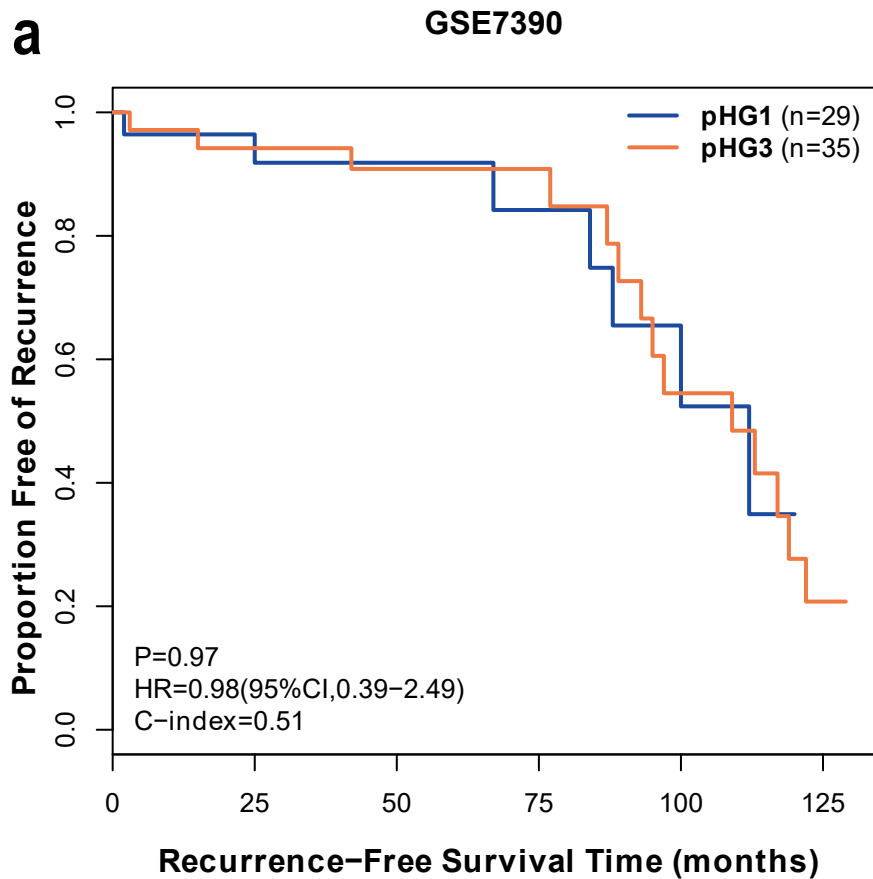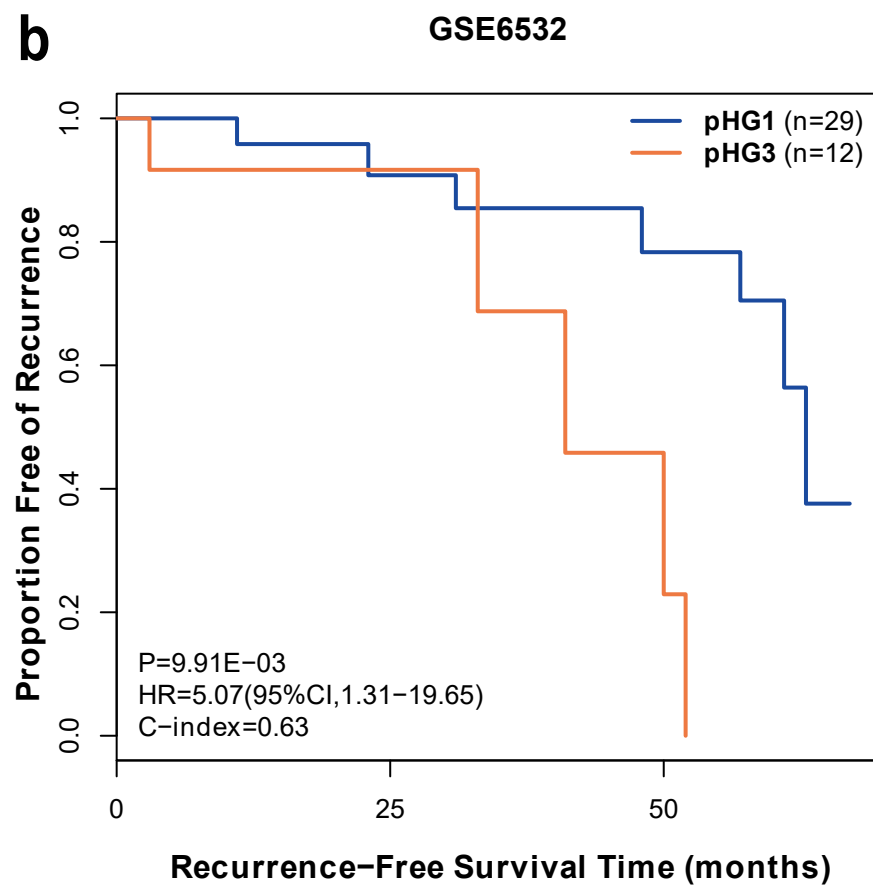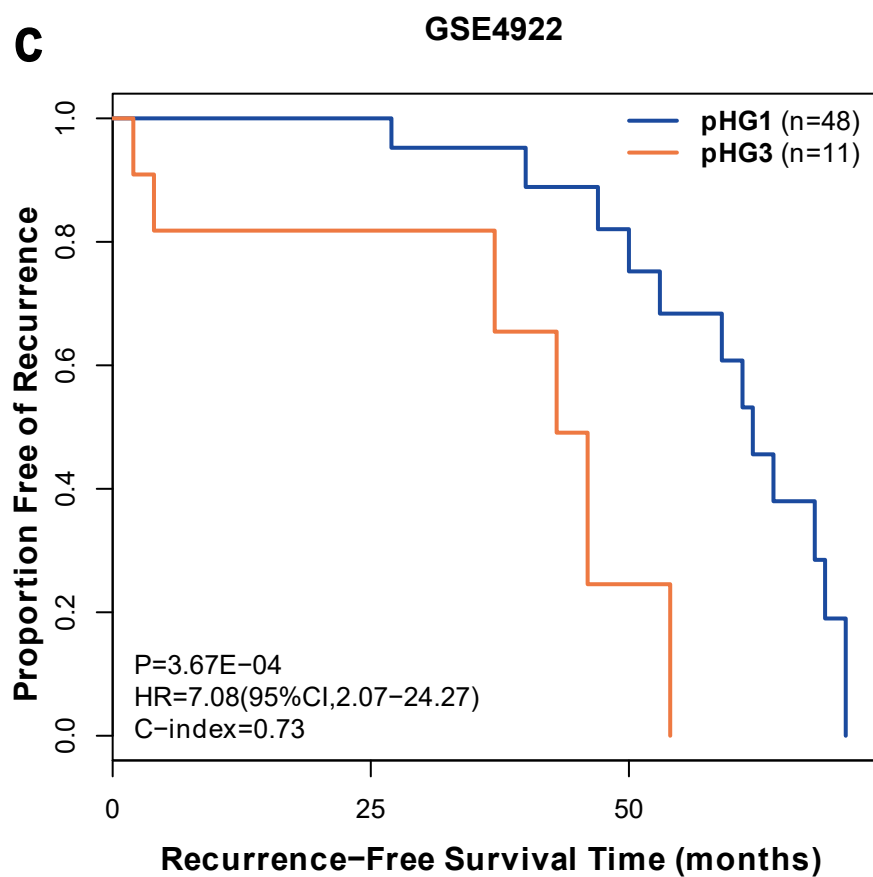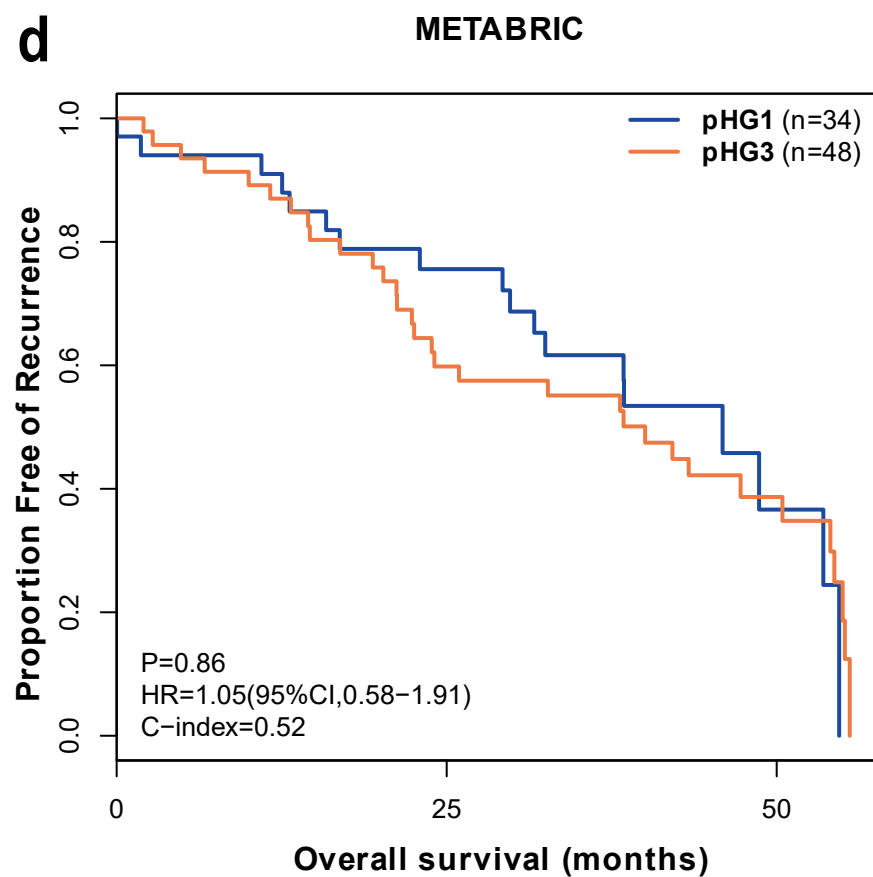

Supplement: Supplementary file 2 — Additional file 2: Fig. S1. Kaplan–Meier estimates of survival. (a-c) Relapse-free survival curves for pHG1 and pHG3 patients in dataset GSE7390, GSE6532 and GSE4922. (d) Overall survival curves for pHG1 and pHG3 patients reclassified from all breast cancer patients in dataset EGA. [file 12864_2020_6659_MOESM2_ESM.pdf]

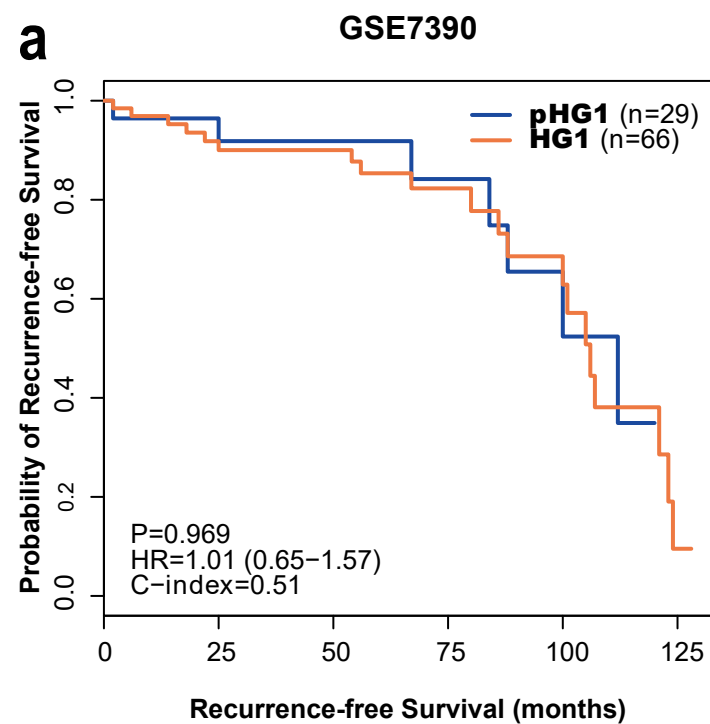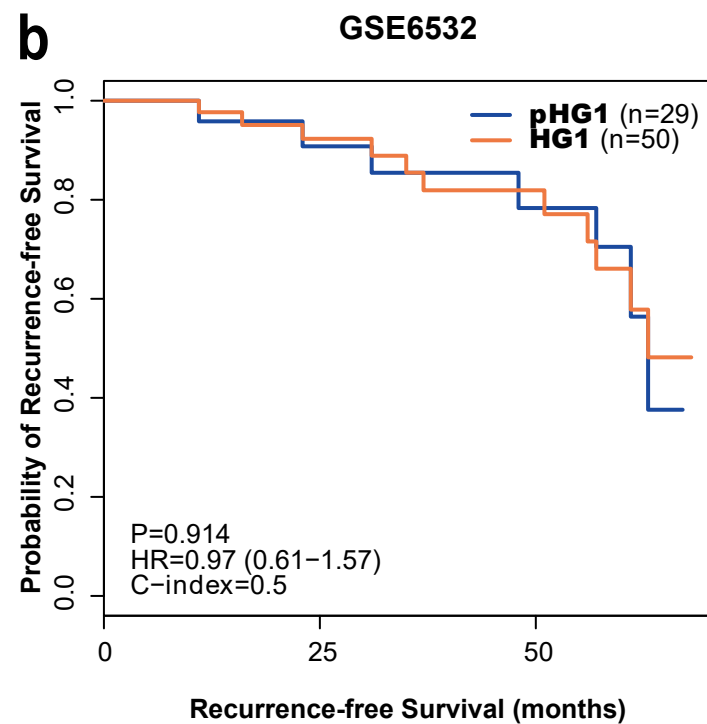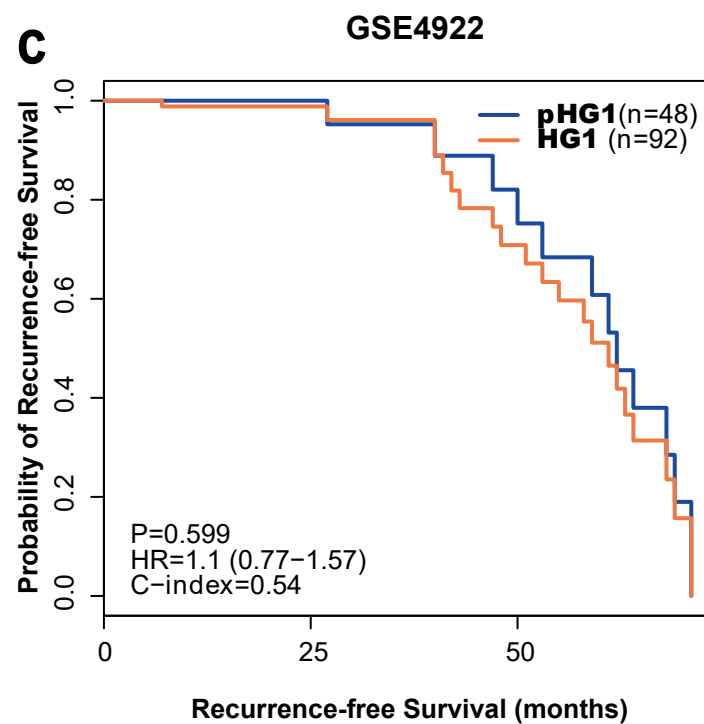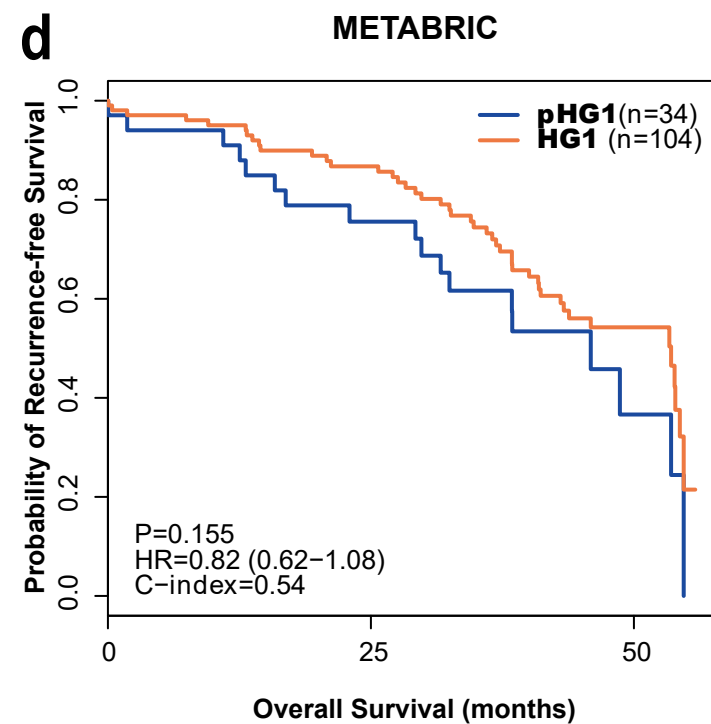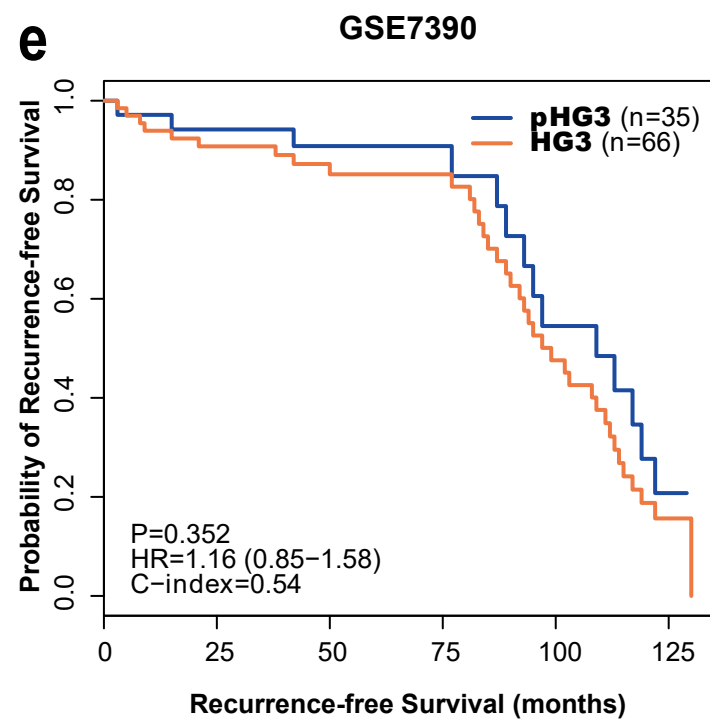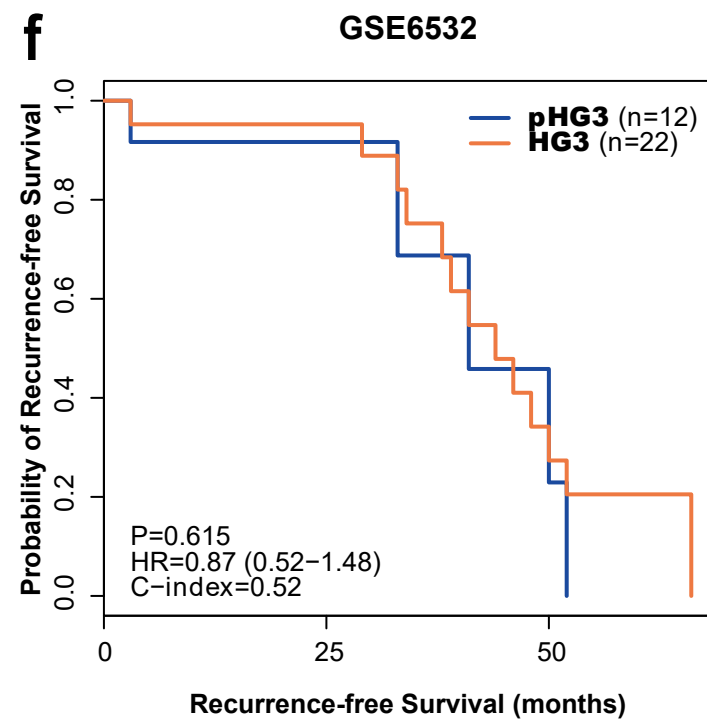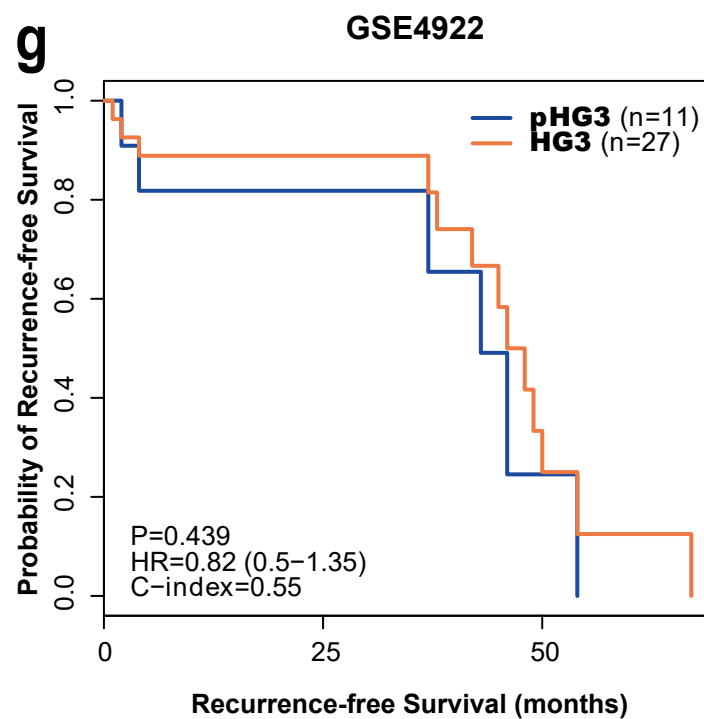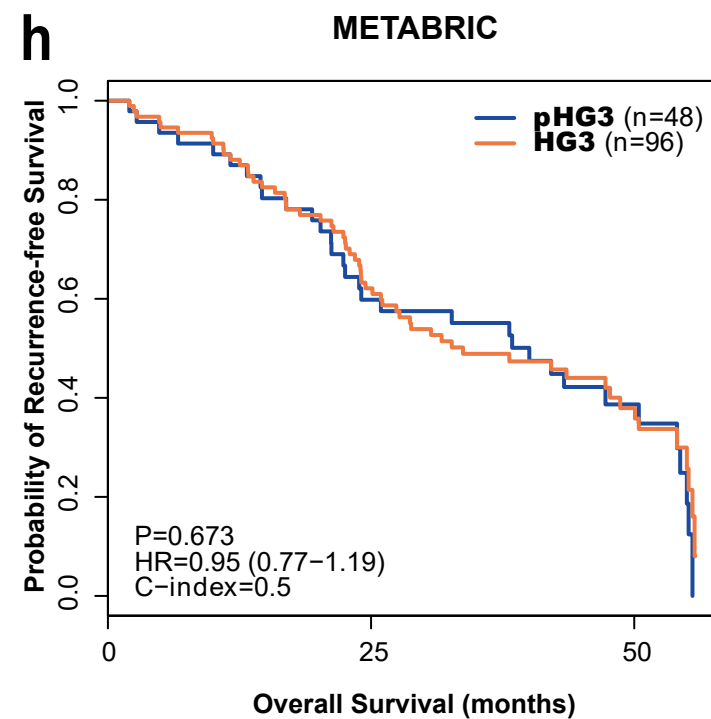

Supplement: Supplementary file 3 — Additional file 3: Fig. S2. Kaplan–Meier estimates of survival. (a-c) Relapse-free survival curves for pHG1 and HG1 patients reclassified from all breast cancer patients in dataset GSE7390, GSE6532 and GSE4922 (d) Overall survival curves for pHG1 and HG1 patients reclassified from all breast cancer patients in dataset EGA. (e-g) Relapse-free survival curves for pHG3 and HG3 patients reclassified from all breast cancer patients in dataset GSE7390, GSE6532 and GSE4922 (h) Overall survival curves for pHG3 and HG3 patients reclassified from all breast cancer patients in dataset EGA. [file 12864_2020_6659_MOESM3_ESM.pdf]

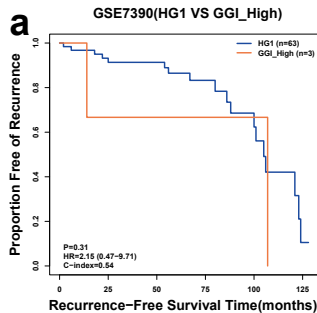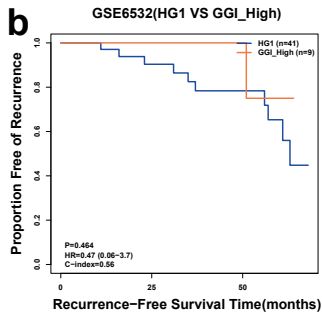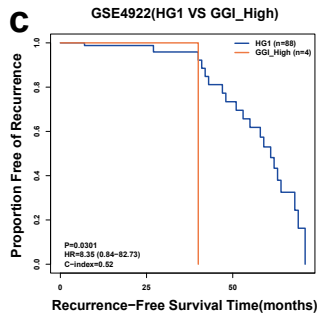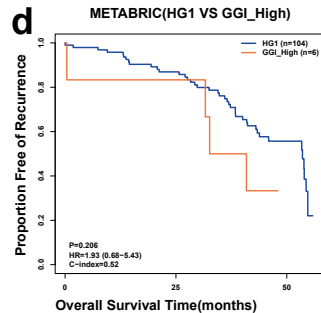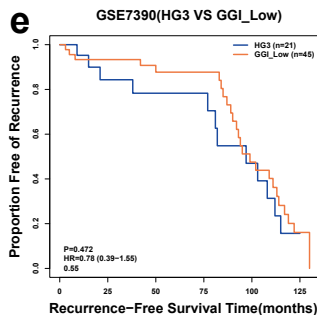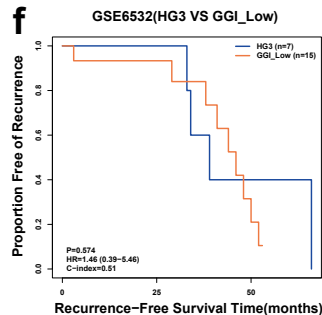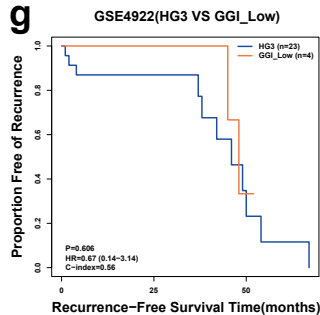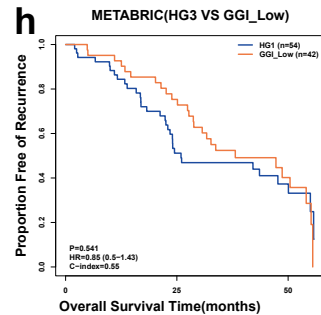

Supplement: Supplementary file 6 — Additional file 6: Fig. S4. Kaplan–Meier estimates of survival (a-c) Relapse-free survival curves for low-risk and high -risk group identified by GGI in HG1 group identified by 10-GPS in GSE7390, GSE6532, GSE4922 (d) Overall survival curves for low-risk and high -risk group identified by GGI in HG1 group identified by 10-GPS in METABRIC (e-g) Relapse-free survival curves for low-risk and high -risk group identified by GGI in HG3 group identified by 10-GPS in GSE7390, GSE6532, GSE4922 (h) Overall survival curves for low-risk and high -risk group identified by GGI in HG3 group identified by 10-GPS in METABRIC. [file 12864_2020_6659_MOESM6_ESM.pdf]

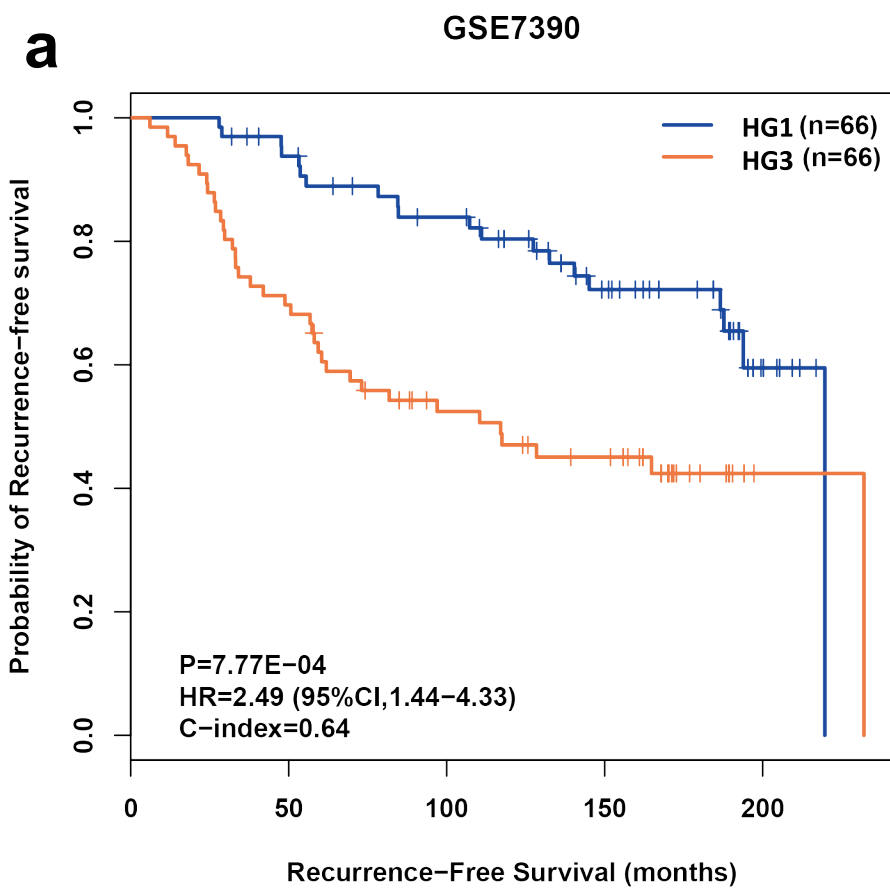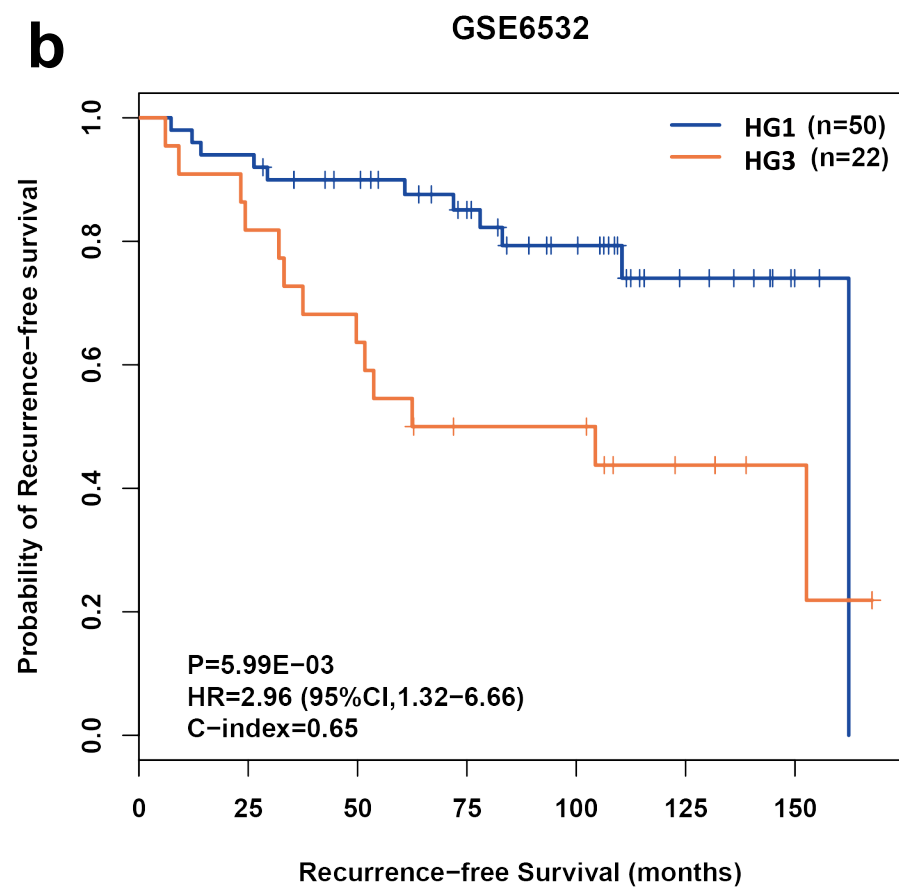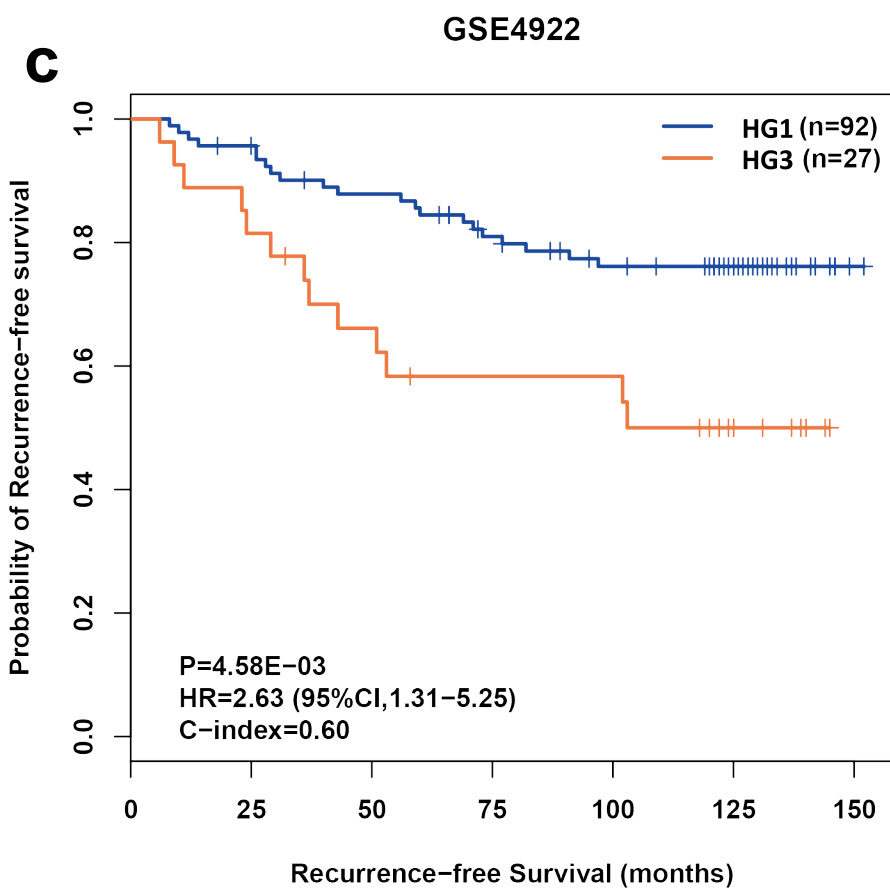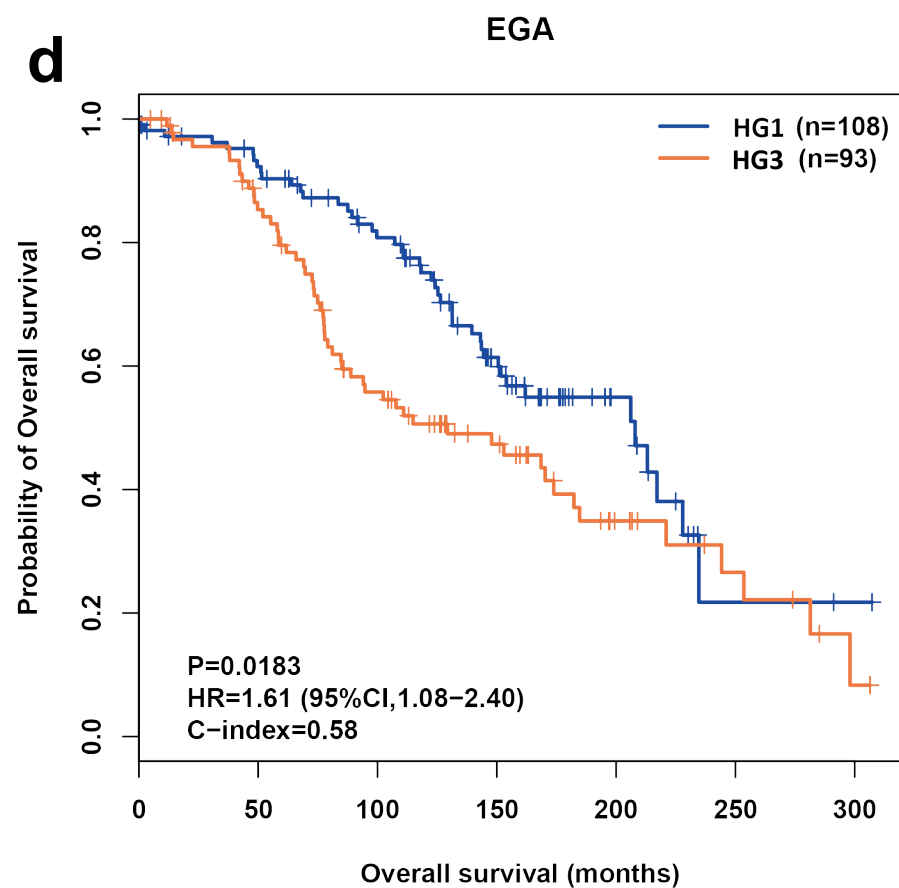

Supplement: Supplementary file 7 — Additional file 7: Fig. S5. Kaplan–Meier estimates of survival. (a) Relapse-free survival curves for HG1 and HG3 patients reclassified from all breast cancer patients in dataset GSE7390. (b) Relapse-free survival curves for HG1 and HG3 patients reclassified from all breast cancer patients in dataset GSE6532. (c) Relapse-free survival curves for HG1 and HG3 patients reclassified from all breast cancer patients in dataset GSE4922. (d) Overall survival curves for HG1 and HG3 patients reclassified from all breast cancer patients in dataset EGA. [file 12864_2020_6659_MOESM7_ESM.pdf]

# GSE16391

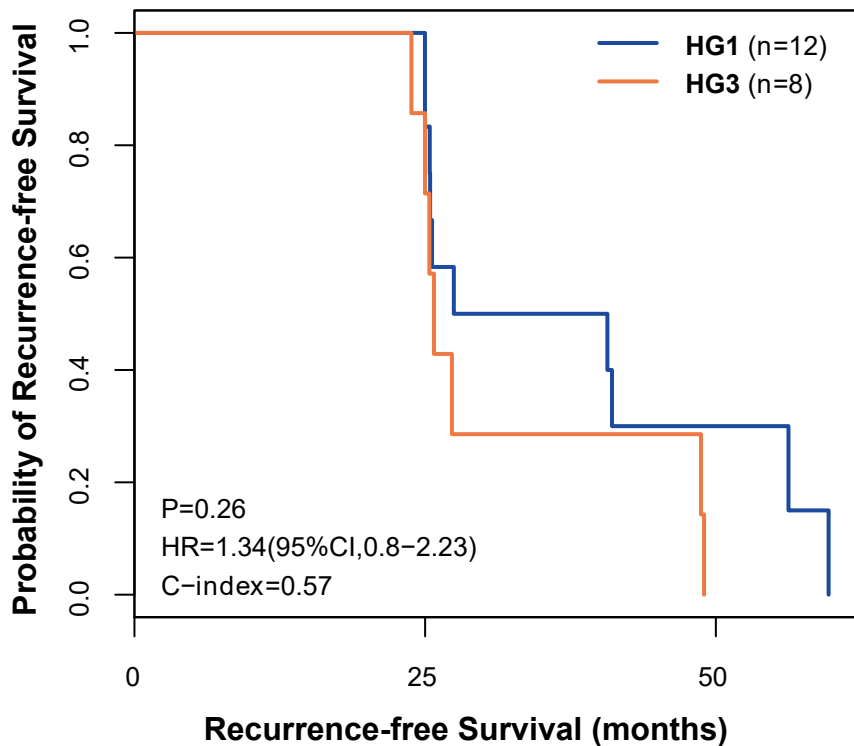

Supplement: Supplementary file 8 — Additional file 8: Fig. S6. Kaplan–Meier estimates of survival. Relapse-free survival curves for HG1 and HG3 patients reclassified from all breast cancer patients in dataset GSE16391. [file 12864_2020_6659_MOESM8_ESM.pdf]
