# Supplementary material for: A qualitative transcriptional signature to reclassify histological grade of ER-positive breast cancer patients
Source: BMC Genomics. 2020 Apr 6;21:283. doi: 10.1186/s12864-020-6659-0 (PMC7132979; doi:10.1186/s12864-020-6659-0)
Supplement: Supplementary file 5 — Additional file 5: Fig. S3. Reproducibility of pathways enriched by differential expressed genes identified in TCGA dataset. The number of times each pathway enriched in the four validation datasets was ranged from 0 to 4. The more positive the number, the deeper the red color of horizontal bar. [file 12864_2020_6659_MOESM5_ESM.pdf]

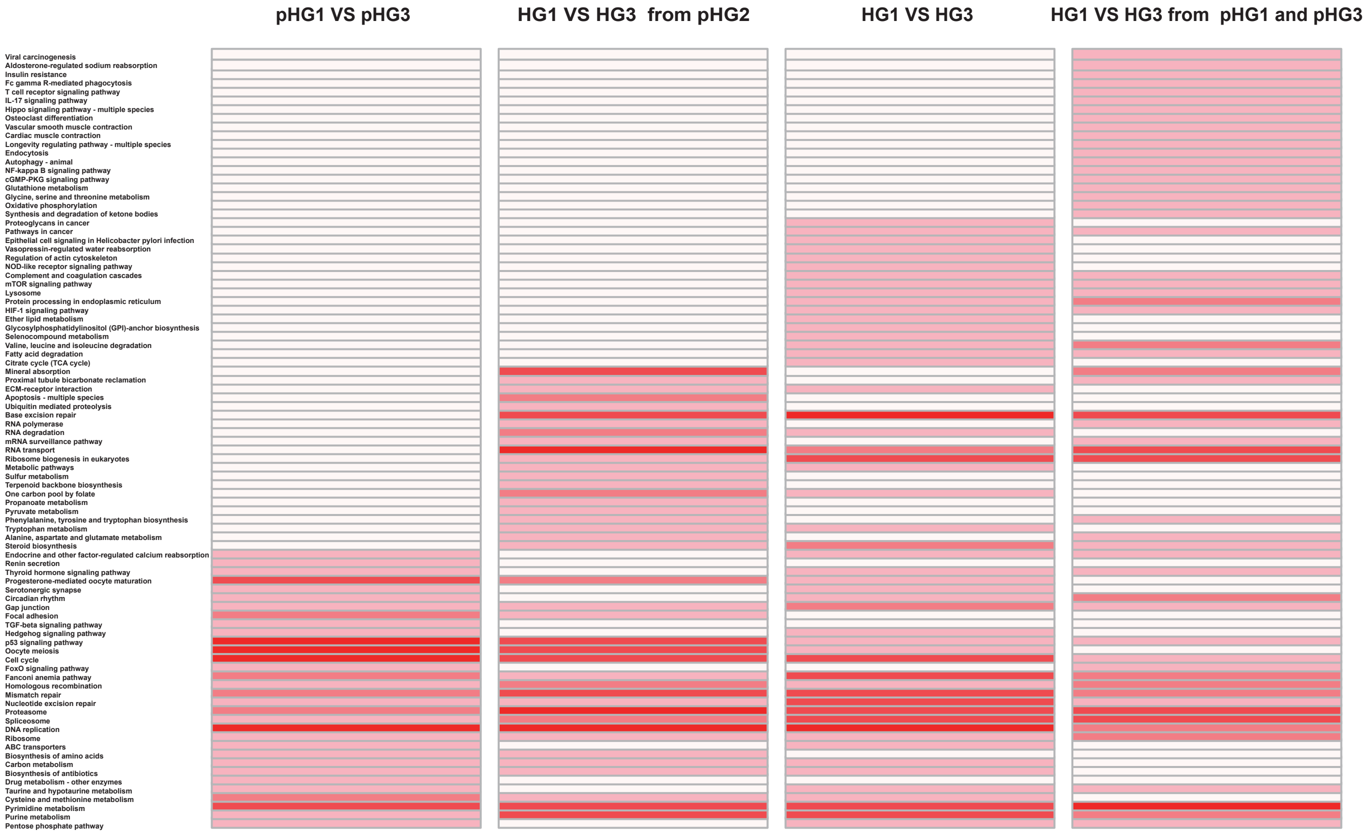
pHG1 VS pHG3
HG1 VS HG3 from pHG2
HG1 VS HG3
HG1 VS HG3 from pHG1 and pHG3
Viral carcinogenesis
Aldosterone-regulated sodium reabsorption
Insulin resistance
Fc gamma R-mediated phagocytosis
T cell receptor signaling pathway
IL-17 signaling pathway
Hippo signaling pathway - multiple species
Osteoclast differentiation
Vascular smooth muscle contraction
Cardiac muscle contraction
Longevity regulating pathway - multiple species
Endocytosis
Autophagy - animal
NF-kappa B signaling pathway
cGMP-PKG signaling pathway
Glutathione metabolism
Glycine, serine and threonine metabolism
Oxidative phosphorylation
Synthesis and degradation of ketone bodies
Proteoglycans in cancer
Pathways in cancer
Epithelial cell signaling in Helicobacter pylori infection
Vasopressin-regulated water reabsorption
Regulation of actin cytoskeleton
NOD-like receptor signaling pathway
Complement and coagulation cascades
mTOR signaling pathway
Lysosome
Protein processing in endoplasmic reticulum
HIF-1 signaling pathway
Ether lipid metabolism
Glycosylphosphatidylinositol (GPI)-anchor biosynthesis
Selenocompound metabolism
Valine, leucine and isoleucine degradation
Fatty acid degradation
Citrate cycle (TCA cycle)
Mineral absorption
Proximal tubule bicarbonate reclamation
ECM-receptor interaction
Apoptosis - multiple species
Ubiquitin mediated proteolysis
Base excision repair
RNA polymerase
RNA degradation
mRNA surveillance pathway
RNA transport
Ribosome biogenesis in eukaryotes
Metabolic pathways
Sulfur metabolism
Terpenoid backbone biosynthesis
One carbon pool by folate
Propanoate metabolism
Pyruvate metabolism
Phenylalanine, tyrosine and tryptophan biosynthesis
Tryptophan metabolism
Alanine, aspartate and glutamate metabolism
Steroid biosynthesis
Endocrine and other factor-regulated calcium reabsorption
Renin secretion
Thyroid hormone signaling pathway
Progesterone-mediated oocyte maturation
Serotonergic synapse
Circadian rhythm
Gap junction
Focal adhesion
TGF-beta signaling pathway
Hedgehog signaling pathway
p53 signaling pathway
Oocyte meiosis
Cell cycle
FoxO signaling pathway
Fanconi anemia pathway
Homologous recombination
Mismatch repair
Nucleotide excision repair
Proteasome
Spliceosome
DNA replication
Ribosome
ABC transporters
Biosynthesis of amino acids
Carbon metabolism
Biosynthesis of antibiotics
Drug metabolism - other enzymes
Taurine and hypotaurine metabolism
Cysteine and methionine metabolism
Pyrimidine metabolism
Purine metabolism
Pentose phosphate pathway
